# A Hybrid Deep Learning Emotion Classification System Using Multimodal Data

**DOI:** 10.3390/s23239333

**Published:** 2023-11-22

**Authors:** Dong-Hwi Kim, Woo-Hyeok Son, Sung-Shin Kwak, Tae-Hyeon Yun, Ji-Hyeok Park, Jae-Dong Lee

**Affiliations:** Department of Computer Science, Dankook University, 152 Jukjeon-ro Campus, Suji-gu, Yongin-si 16890, Republic of Korea; 32190483@dankook.ac.kr (D.-H.K.); 32192206@dankook.ac.kr (W.-H.S.); 32180196@dankook.ac.kr (S.-S.K.); 32192930@dankook.ac.kr (T.-H.Y.); pjh72230235@dankook.ac.kr (J.-H.P.)

**Keywords:** emotion classification, deep learning, multimodal, BERT

## Abstract

This paper proposes a hybrid deep learning emotion classification system (HDECS), a hybrid multimodal deep learning system designed for emotion classification in a specific national language. Emotion classification is important in diverse fields, including tailored corporate services, AI advancement, and more. Additionally, most sentiment classification techniques in speaking situations are based on a single modality: voice, conversational text, vital signs, etc. However, analyzing these data presents challenges because of the variations in vocal intonation, text structures, and the impact of external stimuli on physiological signals. Korean poses challenges in natural language processing, including subject omission and spacing issues. To overcome these challenges and enhance emotion classification performance, this paper presents a case study using Korean multimodal data. The case study model involves retraining two pretrained models, LSTM and CNN, until their predictions on the entire dataset reach an agreement rate exceeding 0.75. Predictions are used to generate emotional sentences appended to script data, which are further processed using BERT for final emotion prediction. The research result is evaluated by using categorical cross-entropy (CCE) to measure the difference between the model’s predictions and actual labels, F1 score, and accuracy. According to the evaluation, the case model outperforms the existing KLUE/roBERTa model with improvements of 0.5 in CCE, 0.09 in accuracy, and 0.11 in F1 score. As a result, the HDECS is expected to perform well not only on Korean multimodal datasets but also on sentiment classification considering the speech characteristics of various languages and regions.

## 1. Introduction

Emotion classification technology offers various advantages, such as emotion-based decision making and improved communication, through the ability of artificial intelligence to think like humans [1]. Recently, research on emotion classification from text has been conducted using the bidirectional encoder representations from the transformer (BERT) model [2,3]. BERT is distinctive for its pretraining on massive text data and its strength in text analysis through bidirectional learning, making it well suited for emotion classification. Text data used in emotion classification primarily include natural language sentences from speech situations, scripts, or written text. However, conventional research that relies on single types of text data, such as BERT, fails to utilize related data from speech situations, resulting in the distortion of the meaning of other data and the inability to account for regional dialects unique to different countries. Therefore, the objective of this paper is to develop a system that addresses these issues and enables versatile emotion classification even in scenarios with limited sample data, such as regional dialects or different languages.

In this paper, a hybrid deep learning emotion classification system (HDECS) using multimodal data is proposed. HDECS processes multimodal data, including human physiology, audio, and script data from speech situations, employing various deep learning models to perform emotion classification, thereby enabling the provision of multiple services to users. The proposed HDECS makes use of LSTM [4] and CNN [5], and two deep learning models to handle multimodal data. The results of these two models, combined with script data, are defined in Section 3.4.4. Emotion classification using the BERT model is then performed on the amalgamated data. Furthermore, traditional natural language processing models perform emotion classification using single-type data. In contrast, the proposed system leverages various multimodal data present in conversation situations, enabling the classification of emotions that are difficult to distinguish using text alone and enhancing the model’s performance. As a result, a more effective approach to emotion classification is demonstrated.

The purpose of this research is to enhance the emotion classification performance of large-scale natural language processing models to address the issues with the existing BERT and to develop a system applicable to various languages. Then, it aims to confirm whether the proposed HDECS enhances emotion classification performance. To achieve this, a CASE study on ‘Korean’, a language that poses challenges in natural language processing due to its flexibility in word order, multiple expressions of the same information, and the absence of differences between declarative and interrogative sentences, is conducted. In the CASE study, the KLUE/roBERTa [6,7] model trained with data suitable for the Korean language is used. To determine the improvement in the performance of the model, the performance of the existing KLUE/roBERTa model is compared with the KLUE/roBERTa model after passing through HDECS based on three functions: accuracy, F1 score [8], and categorical cross-entropy (CCE) [9]. Section 2 explains related works, Section 3 provides a detailed explanation of the HDECS (system architecture, LSTM model, CNN model, cross-LSTM and CNN, and BERT). Finally, Section 4 and Section 5 describe the research results and conclusions.

## 2. Background Study

### 2.1. Emotion Classification

Emotion [10] classification refers to the process of identifying and categorizing emotions from text or voice data using natural language processing and machine learning techniques [11]. In recent times, the increasing prevalence of devices such as smartphones and smartwatches has made it possible to collect speech data from various situations, leading to the proliferation of research and services related to emotion classification [12,13,14,15]. The classification of emotions gathered from platforms like social media [13] is used by businesses to understand individual preferences and tailor their strategies. In the field of healthcare, it aids in the early detection of extreme behaviors and depression, assisting in mental health therapy [14,15]. The advancement of emotion classification technology not only improves the performance of emotion classification models but also enables the provision of various services with enhanced accuracy and reliability.

### 2.2. Deep Learning

Long short-term memory (LSTM) models play a significant role in understanding and exploiting the complex patterns and dependencies in sequence data and are often used to improve data-driven decision making and predictive models. Recent research has utilized sequence data from various fields to solve many problems. Examples include emotion classification from EEG [16], health checks using biometric data [17], and emotion classification [18]. However, LSTM models have difficulty learning in the presence of missing data [19], making it difficult to achieve high accuracy until the missing data are removed or imputed. Therefore, to solve this problem, we used a complementary method using other data.

Convolutional neural network (CNN) models are effectively used to process and extract features from multidimensional data such as images and videos. Typical uses include image recognition for object detection and image classification through computer vision to help autonomous driving [20,21]. They are also used to create waveform images from speech, analyze sound signals, and recognize voice commands. The disadvantage of these CNN models is that they are difficult to design and understand. Many hyperparameters and architectural choices are required to design a CNN model, which can make the initial model selection difficult. Also, since the features and weights are extracted and learned from within the model, the model behavior is difficult to understand and interpret, making it difficult to explain.

### 2.3. Multimodal Data

People perceive the world using different senses, and likewise, artificial intelligence needs to process data from multiple sources to comprehend and engage with the world in a way similar to humans [21]. Recent studies [22,23] aim to use data from different sources to address practical issues and improve the performance of machine learning models. One of the most common approaches is to classify sound and imagery in video data, subsequently extracting and combining information using specialized models for each data type [24]. This method incorporates techniques such as attention mechanisms [25,26] to highlight the interaction between visual and language information. Another way to achieve this is by pulling out key features from each type of data and handling all those features within a single model [27]. This method involves training different data types using different models to extract features. Then, these features are systematically combined, beginning with two kinds of data features and eventually adding three or more types of data features.

The above approaches have improved model performance using either a single model or multiple models for multimodal data. However, it is inconvenient to redesign the entire architecture every time one needs to handle new data types, and deciding which data type to select is challenging. Thus, in this study, we sought to tackle these challenges by using the flexibility of interconnected models.

### 2.4. Emotion Classification Using BERT

BERT is a structure that stacks the encoders of a transformer utilizing an attentional mechanism. In order to use the pretrained BERT for sentiment classification, a fine-tuning process is required to further train the model with emotion-labeled text data.

However, relying solely on scripts for emotion prediction has its limitations. Each type of script data is a single sentence, and predicting emotions from a single sentence can be challenging unless it directly contains emotion-related words. Analyzing contextual relationships and predicting emotions from dialogues, rather than scripts alone, is an option. However, as sentences and conversations grow longer, substantial computational resources are consumed via BERT’s attention mechanisms.

To address the specifics of Korean emotion classification under investigation in this paper, fine-tuning is essential for Korean text in the BERT model. A pretrained BERT model adapted to Korean, pretrained on Korean-appropriate data, is required. The Korean Language Understanding Evaluation (KLUE) provides benchmark data for evaluating natural language understanding in Korean. The KLUE offers two pretrained models: KLUE/BERT, pretrained on KLUE data; and KLUE/roBERTa, pretrained using the roBERTa approach with dynamic masking, increased data, and larger batch sizes, enhancing performance.

## 3. Proposed Hybrid Deep Learning Emotion Classification System

### 3.1. System Architecture and Outstanding Value of Hybrid Deep Learning Emotion Classification System

**Figure 1:**
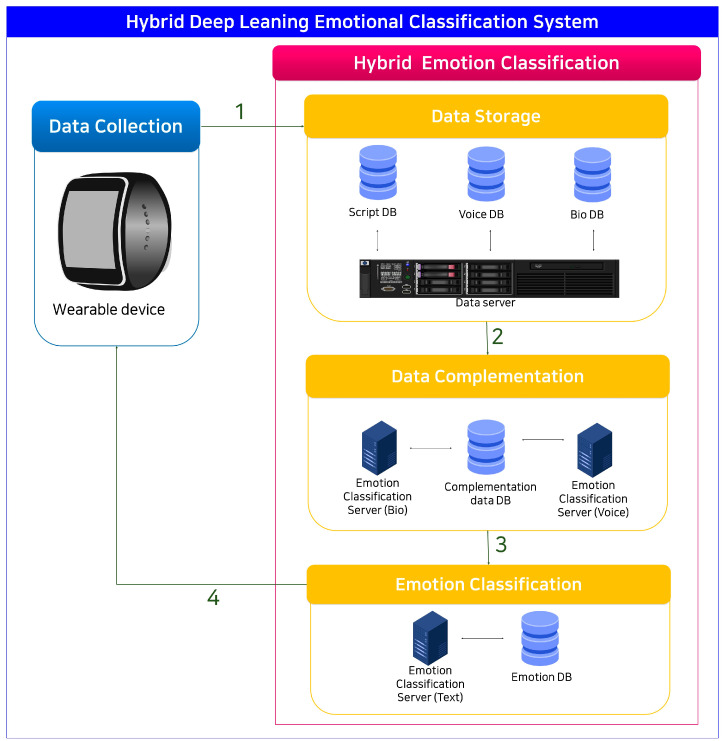
Hybrid deep learning emotion classification system (HDECS).

HDECS is a hybrid deep learning emotion classification system using multimodal data. It collects physiological data, audio, and script data through a customized AI wearable device, and these data go through hybrid multimodal deep learning to provide users with emotion recognition services, as shown in Figure 1.

Figure 1 represents the architecture of the HDECS. The HDECS is composed of a device that collects multimodal data and deep learning for emotion classification. Multimodal data are sent to data storage, where they are separated into biodata, audio data, and script data. Biodata and audio data are then processed using LSTM and CNN models and combined with script data to complement the data. These complemented data are used to classify emotions with the BERT model, thus improving the emotion classification performance of the natural language processing model. Finally, the model has a structure for transmitting classified emotional information to wearable devices.

In contrast to traditional sentiment classification using the standard BERT model, the approach outlined in this paper, known as HDECS (hybrid deep emotion classification system), enriches BERT’s input by including emotion-specific scripts associated with different countries. This unique method offers the capability to classify natural language emotions based on geographic and cultural distinctions.

### 3.2. Dataset

The dataset utilized in this study is the Korean-based multimodal emotional dataset known as the KEMDy20 (Korean Emotional Multimodal Dataset in 2020). The KEMDy20 comprises multimodal emotional data collected for the purpose of examining the relationship between utterance speech and transcripts of utterances, contextual meaning (lexical), and physiological responses. It encompasses data related to signal–skin conductivity (EDA—electrodermal activity), pulse-related information (IBI—inter-beat interval), and wrist skin temperature (TEMP—temperature) in connection with the emotions of two speakers.

### 3.3. Flowchart of Hybrid Deep Learning Emotion Classification System

In the proposed HDECS, the biodata utilized are in the form of time-series data, and due to the presence of missing values in IBI, a prerequisite is to address these gaps. To achieve this, LSTM is employed to predict and fill in the missing IBI data. The audio data are transformed into a spectrogram through a fast Fourier transform. A series of transformations are applied for Mel and Decibel to reflect human auditory characteristics, ultimately resulting in the generation of a log Mel spectrogram in a two-dimensional form for input into the CNN.

**Figure 2:**
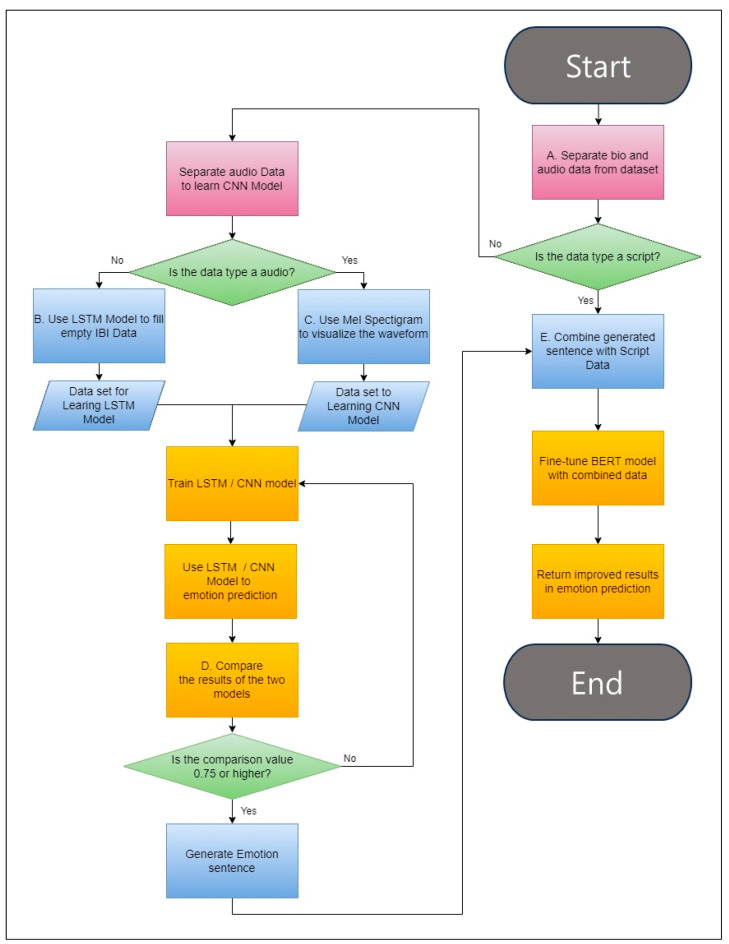
Flowchart of HDECS.

Subsequently, LSTM and CNN are applied to both the biodata and audio data to predict emotions independently. These predictions are then combined, and emotion-related scripts are appended to the script data that will be input to BERT. Through this process, fine-tuning with the BERT model enhances overall performance. The outlined process is illustrated in Figure 2.

Figure 2 shows the flowchart of HDECS. A in Figure 2, multimodal data are separated into speech data and script data. The remaining biodata and audio data undergo data preprocessing to classify emotions using LSTM and CNN, as depicted in B, C in Figure 2.

B in Figure 2, depicts the process of filling in missing values in biodata, including human physiological signals, using an LSTM model, defined in Section 3.4.1. Meanwhile, C in Figure 2 represents the process of visualizing the waveform of audio data in conversational contexts, utilizing Mel spectrograms, defined in Section 3.4.3.

The preprocessed data are then subjected to emotion classification using their respective models, and the results are compared. Iterative training is performed until a threshold (0.75) is exceeded. Subsequently, the results from the cross-LSTM and CNN (CLC) process are combined with speech script data to facilitate transfer learning with BERT, represented in E in Figure 2. This approach combines various types of observed data in conversational scenarios to enhance both accurate emotion classification and validity.

Finally, the trained model undergoes evaluation. HDECS leverages the combination of results from LSTM and CNN to enhance BERT’s accuracy and overcomes the limitations of single-modal data-based emotion classification, contributing to improved performance.

### 3.4. Case of Korean Hybrid Deep Learning Emotion Classification System

#### 3.4.1. Predicting IBI Missing Values Using LSTM Models

Missing values in IBI data can be attributed to various factors, including weak heartbeats or external shocks during data collection. Furthermore, gaps in the periods of IBI data may arise due to differences in data acquisition durations compared to other biosignals. The challenge of managing such data with missing values is addressed in this paper; the process of imputing missing values in these data is described using Algorithm 1, with the corresponding parameters detailed in Table 1.

In Table 1, the “TotalTime” signifies the aggregate duration of the input data, segmented into dil units. The time span extending from the present moment to the most recent time slot containing an IBI value is denoted as “pre_time”, while the interval from the first time slot featuring an IBI value after the current time is termed “post_time”. The discrepancy between “post_time” and “pre_time” is defined as “interval_time”, and the variations in IBI values for the respective time slots are designated as “interval_bio”.
**Algorithm 1:** Data_complementationInputBioOutputresult_model1.Def Data_complementation(bio):2.       Interpolated_bioes = []3.       For time in TotalTime:4.             if post_time = pre_time:5.                   Interpolated_bio = bio[post_time]6.             else:7.                   Interpolated_bio = bio[pre_time]+(dil-pre_time)*(Interval_bio)/(Interval_time)8.             Interpolated_bioes.append(Interpolated_bio)9.       Return Interpolated_bioes10.Use Algorithm 2 (Lstm model)11.return result_model

Lines 1 through 9 of Algorithm 1 delineate the “Data_complementation” function, which has been developed to address the issue of missing values in IBI data. This function employs linear interpolation to bridge the gaps or missing data points between the recorded measurement times. In line 7, a linear connection is established between values at “pre_time” and “post_time”, subsequently assigning values corresponding to the unit time of the absent data.

Following this, the outcomes generated by the “Data_complementation” function are harnessed to predict short-term IBI for the entire measurement duration, as depicted in lines 10 to 11. In this instance, an LSTM model is employed, which adheres to a structure akin to that presented in Algorithm 2 and makes use of the parameters delineated in Table 2.

The values denoted as “Metrics_x” in Table 2 are derived through the utilization of weights (W_x) and a bias (b_x) for the computation of a weighted sum. This summation process involves taking the previous time’s hidden state and the current input value, represented as “dfm”, and transforming these components into a matrix.
**Algorithm 2:** LSTM modelInputdfmOutputPred_res1.Initialization weights and biases2.Metrics_x = (W_x*[hst-1, dfm[t]] + b_x)3.for s from 1 to seq:4.       Forget Gate: $fg_{t}$=$\frac{1}{\left(1+e^{-Metrics\_f}\right)}$5.       Input Gate: $ig_{t}$=$\frac{1}{\left(1+e^{-Metrics\_i}\right)}\times \frac{\left(e^{Metrics\_i}-e^{Metrics\_i}\right)}{\left(e^{Metrics\_i}+e^{Metrics\_i}\right)}$6.       Cell State: cst = fgt*cst-1 + igt7.       Output Gate: ogt = $\frac{1}{\left(1+e^{-Metrics\_o}\right)}*\frac{\left(e^{cs_{t}}-e^{cs_{t}}\right)}{\left(e^{cs_{t}}+e^{cs_{t}}\right)}$= 8.       pred_res = $\frac{e^{hs_{t}}}{\sum_{k=1}^{7}e^{hs_{t}}}$9.return pred_res

**Figure 3:**
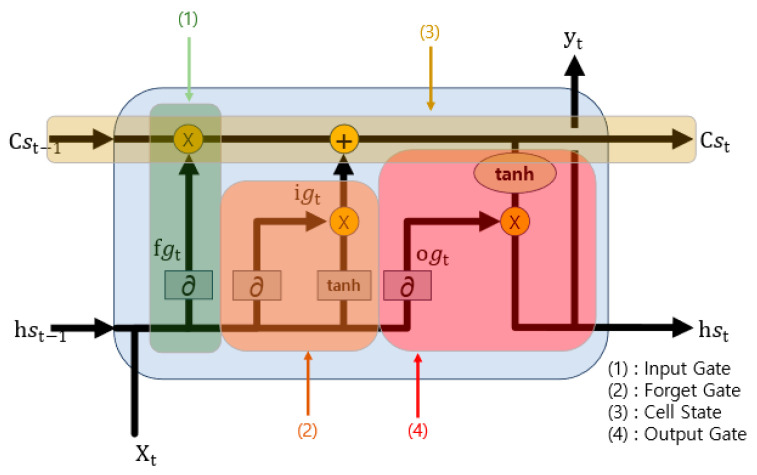
Structure of LSTM.

**Figure 4:**
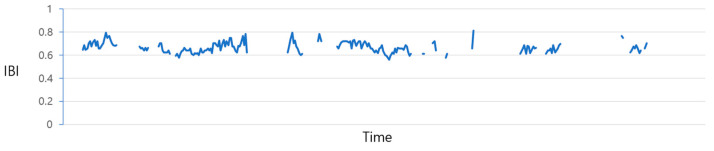
IBI distribution chart before forecast.

**Figure 5:**
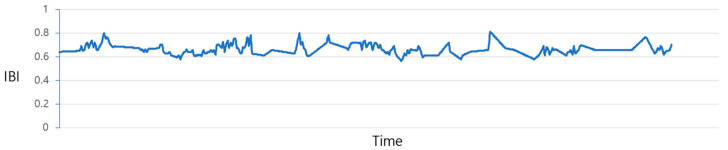
IBI distribution chart after resampling.

**Figure 6:**
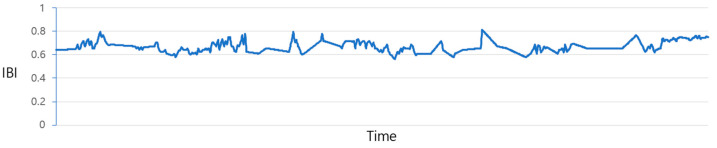
IBI distribution chart after forecasting.

The LSTM model utilized for the prediction of missing values adheres to Algorithm 2 and is visually represented in Figure 3. Algorithm 2 iterates a specified number of times, denoted as “seq”. In line 4, the forget gate, which is akin to element (1) in Figure 3, plays a crucial role. It employs a sigmoid function to assess the importance and, based on this evaluation, determines how much of the previous state to consider. Line 5 corresponds to the input gate, which is like element (2) in Figure 3. This gate employs a sigmoid function to determine the proportion of the input to include and subsequently updates the cell state in line 6 by amalgamating the input data that have been processed through the hyperbolic tangent (tanh) function. The resultant cell gate is analogous to element (3) in Figure 3. Line 7 pertains to the output gate, which is responsible for generating the final output, equivalent to element (4) in Figure 3. This gate, after passing through the Softmax function, produces the ultimate output. This algorithm is implemented as a model with a structure closely resembling that described in Table 3, specifically designed for the purpose of imputing missing values. The process of missing value imputation is visually depicted in Figure 4, Figure 5 and Figure 6.

Table 3 delineates the configuration of the LSTM model employed in Algorithm 1. This model comprises a solitary LSTM layer utilized for prediction. Following this, it traverses a dropout layer, implemented to mitigate the risk of overfitting. Subsequently, the data are processed through a Softmax layer, culminating in the generation of the ultimate output.

Figure 4 illustrates the plot of IBI values before the prediction process. Due to the irregular data measurement intervals, as previously discussed, there exist gaps where values are missing. The process of filling in these gaps is illustrated in Figure 5 and Figure 6. The data from Figure 4 are adjusted utilizing the “Data_complementation” function, as defined in line 1 of Algorithm 1. This transformation aligns the intervals between data points with those of other biodata and rectifies missing values stemming from variations in time intervals. Figure 5 depicts the graph of the data after undergoing this adjustment. Nevertheless, there are still time intervals without IBI values, even after this procedure.

In such instances, applying the same missing value imputation method shown in Figure 6 becomes challenging because of the insufficient data available to adequately supplement the missing values. Consequently, predictions are generated by employing the LSTM model outlined in line 10 of Algorithm 1. Figure 6 represents the distribution of the results obtained through LSTM predictions.

#### 3.4.2. Emotion Classification Using LSTM Model

Emotion classification was carried out using biodata that were enhanced through IBI prediction. The model employed for this task is akin to Algorithm 2. The entire dataset was partitioned into training, validation, and test datasets in an 8:1:1 ratio, ensuring an equitable distribution of participants, primarily intended for training purposes. About 13,000 biofiles were extracted from KEMDy20 conversation situations and were divided after preprocessing. The training process encompassed the random extraction of 20 samples per class from the training dataset, and this operation was iterated three times, resulting in a total of 1600 iterations. With respect to the conditions specified, the trained model accomplished an accuracy of 0.82.

#### 3.4.3. Mel Spectrogram CNN

The Mel spectrogram [28] is a technique used to analyze audio data by applying the Fourier transform [29] to them. These data are then converted to the Mel scale [30], considering the pitch variations in human speech. To reduce information loss, audio data undergo this Mel scale transformation and are further subject to a logarithmic transformation, resulting in a log Mel spectrogram [31]. Table 4 below lists a detailed explanation of the key parameters in Algorithm 3.

The STFT function in Table 4 performs the short-time Fourier transform [32], which is calculated on the original audio data using the parameters n_fft and hop_len, where n_fft stands for the frame length, and hop_len indicates how much the frames overlap. This function analyzes the audio signal in the frequency domain to generate the spectrogram, stft_sp. Algorithm 3 below represents the process of converting speech files into log Mel spectrograms. The input for Algorithm 3 includes wavs, n_fft, h_len, and n_mels, and the output is padded_mels.
**Algorithm 3:** Make Mel scale spectrogram and pad from speech filesInputwaves, num_fft, hop_len, num_melsOutputlog_mels1.for each wav in waves:2.       stft_sp = STFT(wav, num_fft, hop_len)3.       convert stft_sp to mel_sp using the mel_filters with num_mels4.       log_mel = 10*log10stft_sp’s power5.       add log_mel to log_mels6.find the longest column length in the log_mels7.pad column with zeros to the longest column length8.return log_mels

(1) $$\mathrm{STFT}\left\{x\left[n\right]\right\}\left(m,\omega \right)\equiv X\left(m,\omega \right)=\Sigma_{n=-\infty }^{\infty }x\left[n\right]w\left[n-m\right]e^{-i\omega n}$$

(2) $$w\left(n\right)=0.5\left(1-\cos\left(\frac{2\pi k}{N}\right)\right)$$

(3) $$\mathrm{spectrogram}\;\left\{x\left(t\right)\right\}\left(\tau ,\omega \right)\equiv |X\left(\tau ,\omega \right){|}^{2}$$

To conduct STFT(1), the audio file is first split into frames of a consistent length determined by num_fft. These frames have intervals set using hop_len to ensure they overlap. Then, a Hamming window function (2) is applied to each frame. This function [33] helps maintain continuity, enhances specific frequency domains, and diminishes others.

Lines 1 to 5 explain how audio files are transformed into log Mel spectrograms. In line 2, the STFT function is applied to the original signal x along with parameters num_fft, hop_len, and num_mels, performing a local Fourier transformation. For this, num_mels sets the number of Mel filters, which controls how finely the frequency domain is divided in the Mel scale. In line 3, mel_filters represents the Mel filter bank [34]. This filter bank is visualized as triangular shapes in the frequency domain. These triangular filters encapsulate specific frequency ranges and are employed to extract energy within corresponding segments of the frequency spectrum. The shape and size of the Mel filter bank are determined based on the sampling rate of the input data and the desired frequency range representation in the Mel scale. So, in lines 2 to 3, the STFT function produces the spectrogram stft_sp, and the Mel spectrogram mel_sp, which captures human auditory characteristics, is acquired by employing a Mel filter bank. In line 4, the power of the Mel spectrogram mel_sp is obtained using Equation (3), and then it is transformed into the log_mel representation in Decibel units [35].

The log Mel spectrogram data from the audio file are represented as a 2D matrix. Notably, n_mels, the number of Mel filters, determines the fixed number of rows, while the number of columns varies depending on the length of the original audio data. As explained in lines 6 to 7, zero padding is carried out, where any empty spaces are filled with zeros.

**Figure 7:**
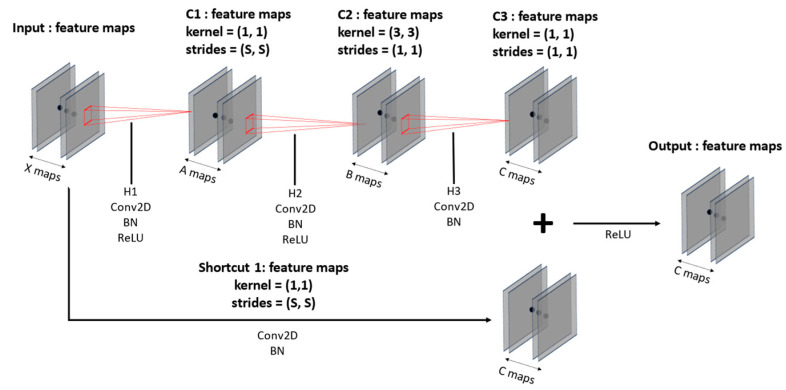
Shape of convolution block with filter (A, B, and C) and strides (S).

**Figure 8:**
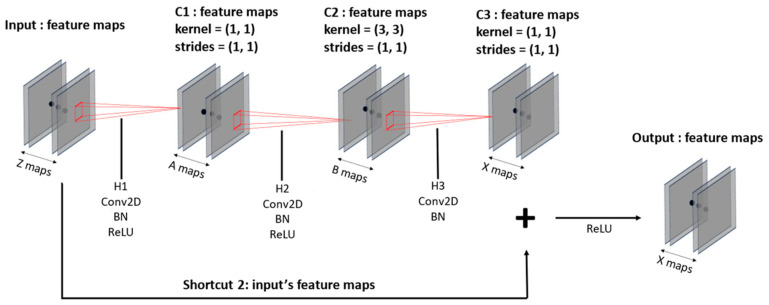
Shape of identity block with filter (A, B, and C).

The input data with a fixed size are trained with a CNN-based ResNet-50 model, as shown in Table 5, along with Figure 7 and Figure 8. The ResNet-50 used here reduces the size of feature maps and increases their numbers as the layers deepen. Moreover, the model structure in Figure 7 and Figure 8 incorporates shortcut connections, which serve to prevent overfitting and gradient vanishing as the layers grow deeper. This design also leads to faster training and superior performance compared to earlier versions of ResNet models.

The model for training audio data consists of a CNN-based ResNet-50 [36] architecture, which includes convolution blocks, identity blocks, pooling layers, global average pooling (GAP) layers [37], and fully connected (dense) layers for classification, as shown in Table 5.

Figure 7 illustrates the convolution block and Figure 8 shows the identity block. Both blocks compress the number of input feature maps in the initial convolution layers and then expand it back to the initial number of a 1 × 1 kernel; the second layer employs a 3 × 3 kernel; and the third layer returns to a 1 × 1 kernel, forming a deep neural network. This kernel configuration, shown in Figure 7 and Figure 8, allows for the construction of deeper neural networks with reduced training time compared to the original ResNet. The convolution blocks can flexibly set strides to increase or decrease the size of feature maps, and identity blocks leverage the fact that input and output sizes remain the same to construct deep layers. Batch normalization [38] is applied to each layer following the convolution layers to normalize the distribution of input data, enhancing training stability and speed. Additionally, a GAP layer is used to flatten 3D data into 1D data and reduce spatial information loss. With the utilization of these spatial features, the model learns and comprehends spatial information more effectively.

The audio data are transformed into spectrograms and pass through ResNet-50, as described in Table 5. Throughout this process, the data that initially had a single feature map exhibit a reduction in feature map size as they are processed through the deeper layers, leading to faster processing. However, the number of feature maps increases, allowing the model to learn more detailed spatial information. Because of these characteristics, the model can reduce input data size while maintaining high accuracy on the dataset. Furthermore, it addresses issues that arise with deepening layers, such as overfitting and gradient vanishing, through shortcut connections.

Table 6 presents the values used when transforming audio data into log Mel spectrograms. The value for the padding size used in this context corresponds to the max_length obtained in Algorithm 3.

The CNN model is trained on the entire dataset, which was partitioned with an equal distribution of participants and divided into training, validation, and test sets at an 8:1:1 ratio. About 13,000 voice files were extracted from KEMDy20 conversation situations and were divided after preprocessing. The training process involves randomly extracting 20 samples per class from the training dataset and repeating this process three times, for a total of 1600 iterations. Under the specified conditions in Table 6, the trained model achieved an accuracy of 0.772.

#### 3.4.4. Cross-LSTM and CNN

To incorporate the results from Section 3.4.2 and Section 3.4.3 [39] into the scripts by adding sentences that convey emotions, Algorithm 4 below is employed. Table 7 below outlines the parameters of Algorithm 4. The input for Algorithm 3 includes P_lstm_ and P_cnn_, and the output is emo_scripts.
**Algorithm 4:** Cross-LSTM and CNN (CLC)InputP_lstm_, P_cnn_, scriptsOutputemo_scripts1.pre_emo = [ ]2.for each p_lstm_, p_cnn_ in ZIP(P_lstm_, P_cnn_):3.       if p_lstm_ and p_cnn_ are same:4.             add p_lstm_ to pre_emo5.       else:6.             add the higher-scoring result between p_lstm_ and p_cnn_ to pre_emo7.convert the emotions in pre_emo into emotional sentences8.emo_scripts = scripts + pre_emo9.return emo_scripts

In line 1, a list is created to compare and store the prediction results of two models P_lstm_ and P_cnn_. In lines 2 to 6, identical emotion predictions from P_lstm_ and P_cnn_ are directly appended to the pre_emo list. For different emotion predictions, the model selects the emotion with the higher score between p_lstm_ and p_cnn_ and stores it in pre_emo. Finally, in lines 7 to 9, sentences are generated from the emotion classification results and then appended to the original script. The emotional expression sentences are based on Table 8 below, and the inclusion of these emotional expression sentences in the script serves to complement the model’s emotional classification performance.

Using the threshold values in CLC to derive results is significant. This is because it takes into account the results predicted using different types of data. Disregarding this aspect and immediately aggregating results significantly reduce the reliability of the modified data, leading to a decrease in the final model’s performance. For these reasons, to reflect the characteristics of different data, each result is compared, and, in order to enhance the meaningful performance of BERT, cross-validation is performed to calculate the threshold values as follows: First, the entire dataset is divided into 10 equal portions. For these 10 divided datasets, if the results obtained with CLC improve BERT’s performance, training is stopped, and accuracy is assessed. Finally, an average of accuracy is calculated and used. The value used in this paper’s CLC was 0.75.

In the experiments, sentences expressing emotions intuitively, as presented in Table 8, were integrated. Depending on the specific model and its application, other sentence forms may be utilized. These sentences were predominantly tailored for emotion classification. The emotion data predicted with CLC exhibited an average emotion prediction accuracy of 0.926 when compared to the entire set of emotion labels.

#### 3.4.5. BERT’s Input Complement

In this paper, the learning approach is modified by implementing BERT’s dynamic masking technique and adopting a pretrained roBERTa model with an increased batch size and a more extensive training dataset. This choice aligns with the strengths of roBERTa models [40] for emotion classification compared to various BERT-based models. To assess the performance of a Korean-specific model using Korean as one of the languages, the KLUE/roBERTa-base model, pretrained with the KLUE dataset, is employed.

**Figure 9:**
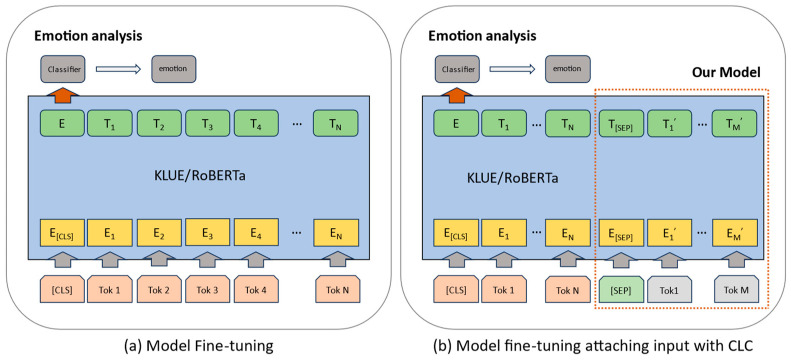
Fine-tuning KLUE/roBERTa and KLUE/roBERTa attaching input for emotion classification.

In the HDECS approach, usable data are initially separated from challenging biodata and audio data. Subsequently, complementary data for the script are generated by applying emotion classification algorithms customized for biodata and audio data. The audio data are subjected to visualization using the Mel spectrogram algorithm, and emotion classification is executed using a CNN-based ResNet model. Regarding biodata, any missing values in IBI are addressed using the IBI prediction LSTM model (IPL). Subsequently, the emotion classification LSTM model (EPL) is used for emotion classification. The outcomes from both models are compared to engage in the cross-LSTM and CNN (CLC) process to generate emotion-related scripts. These scripts are appended to the existing scripts and combined with the KLUE/roBERTa model to enhance model performance, as depicted in Figure 9.

Figure 9a indicates the fine-tuning of emotion-labeled scripts on the KLUE/roBERTa model. In contrast, Figure 9b, as proposed in this paper, illustrates the process through which emotion-related scripts are generated using CLC and added to the existing scripts. Subsequently, fine-tuning is carried out.

The traditional BERT model processes parsed script data, commencing with the [CLS] token to designate the beginning of a sentence. It computes the vector values of the embeddings for each token and produces the token with the highest score in the model. These tokens are concatenated to construct sentences, and the resulting sentences undergo a classification process to predict the corresponding emotion for each sentiment.

To facilitate sentiment analysis with the KLUE/roBERTa model, fine-tuning with emotion-labeled text data is required, followed by transfer learning. For transfer learning, scripts must be tokenized using the byte-pair encoding (BPE) to conform to KLUE/roBERTa’s input specifications. In Figure 9b, the process commences with the [CLS] token, followed by listing tokenized subwords and inserting [SEP] tokens for sentence concatenation. Subsequently, CLC generates the newly added scripts, tokenizes them into subwords using BPE, and appends them. The input is then padded to the maximum token length of 70 tokens, and emotions (neutral, happy, angry, surprised, sad, disgust, and fear) are one-hot encoded to serve as labels, forming the input for the model.

## 4. Experiments and Research

To evaluate the performance of the proposed HDECS, experiments were conducted, and various metrics, including accuracy, precision, and error, were computed. In the experimental setup, high accuracy and precision are indicative of the model’s predictive precision, while a high error value signals inaccuracies in the model’s predictions. Metrics such as accuracy, F1 score, and categorical cross-entropy (CCE) yield values within the range of 0 to 1.

Accuracy quantifies the ratio of correctly predicted emotions to the actual emotions, while the F1 score calculates the harmonic mean of precision, reflecting the accuracy of the model’s predictions and recall, which measures how well the actual correct answers are identified. This metric is effective for handling imbalanced data. When labels are assigned to each emotion, one-hot encoding was utilized to intuitively represent features independently, without implying any order between categories, with the goal of reducing noise.

Categorical cross-entropy was chosen as the loss function due to the nature of the task, which involves multiclass classification, and in low-noise environments, CCE typically converges to higher values. The formula is specified in Table 9, where ‘t’ represents the correct emotion label, ‘y’ represents the predicted emotion label, and ‘C’ signifies the number of emotion classes, with separate calculations for each.

Several techniques were applied to both the KLUE/roBERTa-base model and the proposed HDECS model based on KLUE/roBERTa-base to prevent overfitting and improve model performance. These techniques included the utilization of dropout, early stopping with categorical cross-entropy (CCE) loss, and fine-tuning.

Dropout is a method where neurons within a neural network are randomly deactivated, reducing model complexity and enhancing generalization performance. Initial learning rates were adjusted to control the magnitude of weight updates, ensuring stable training. CCE loss is a commonly used loss function in multiclass classification tasks, and early stopping was applied to the loss function, allowing for the training process to halt when the loss on the validation data no longer decreased, thus preventing overfitting. This approach ensured that the model did not become overly specialized to the training data.

In the experimental setup, a dropout rate of 0.5 was set, and the optimization algorithm Rectified Adam was employed for its fast convergence and stability. The initial learning rate was set at 1 × 10^−5^, with measures in place to ensure it did not fall below this value. During training, CCE loss was implemented, and early stopping was conducted when the loss ceased to decrease.

**Figure 10:**
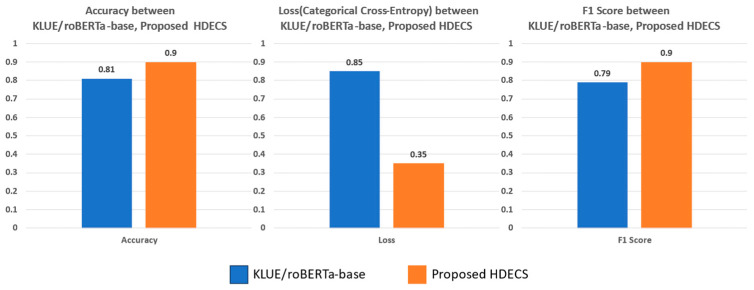
Accuracy, loss, F1 score between KLUE/roBERTa-base, proposed HDECS.

Subsequently, the entire dataset was partitioned into training, validation, and test data using an 8:1:1 ratio for the training and performance evaluation of both the KLUE/roBERTa-base model and the proposed HDECS model based on KLUE/roBERTa-base. In order to maintain a similar class distribution ratio before and after classification, data were classified, considering the substantial differences in class distribution within emotion data samples. The proposed system, which integrated emotion-related scripts generated from CLC results, underwent transfer learning on KLUE/roBERTa-base, yielding performance results that are presented in Figure 10 for the test data.

As shown in Table 10, the accuracy is 0.81 for the KLUE/roBERTa-base and 0.9 for the proposed system, which is 0.09 higher in the proposed system than in the conventional model. Additionally, the loss value using CCE is 0.85 for the conventional KLUE/roBERTa-base and 0.35 for the proposed system, which is lower in the proposed system with a difference of 0.5. The F1 score is 0.79 for the KLUE/roBERTa-base and 0.90 for the proposed system, a difference of 0.11, indicating that the proposed system is also stronger when dealing with imbalances in the same data. Summarizing all the results, It is evident that the accuracy and F1 score are improved, while the loss is reduced, resulting in an enhanced performance in sentiment classification.

## 5. Conclusions and Future Works

This paper introduces a hybrid deep learning emotion classification system (HDECS) using multimodal data. The proposed system processes multimodal data collected from wearable devices through deep learning models and enhances the performance of emotion classification models. The multimodal data consist of biodata, audio data, script data, and emotion evaluation labels. Biodata are analyzed using an LSTM model specialized in learning and predicting time-series data. Additionally, audio data transformed into log Mel spectrograms are processed using a CNN model specialized in analyzing image data. HDECS performs emotion classification with multimodal data, combining the results of deep learning models with script data, and adding this to the input of BERT for emotion classification.

The original KLUE/roBERTa model used for the case study in this paper simply fine-tunes the emotion labeling of textual data. In contrast, the KULE/roBERTa model trained with the proposed system enhances emotion classification performance by combining emotion expression sentences generated from LSTM and CNN models with script data. To evaluate the performance of the proposed HDECS, the accuracy, F1 score, and CCE for emotion-specific validation are used. The results show that the existing model achieves an accuracy of 0.81, while the proposed system achieves an accuracy of 0.90. According to the F1 score, the existing model achieves 0.79, and HDECS achieves an accuracy of 0.90. The CCE loss function shows that the existing model has an error of 0.58, while HDECS has an error of 0.35. Overall, HDECS outperforms the existing model with the highest accuracy of 0.90. This represents significant results in performing emotion classification using multimodal data, particularly in situations where performing emotion classification with only textual data is challenging, demonstrating the potential of HDECS to address such issues. Furthermore, these results signify an important advancement, especially for languages like Korean, where natural language processing is complex.

In future studies, diverse data such as audio, images, and others can be added to HDECS to enhance accuracy instead of adding sentences to the script. Moreover, HDECS can be utilized by developing new models that employ different deep learning approaches, such as an embedding module that uses embedding models and attention functions [41], or ensemble techniques that combine multiple models [42]. Additionally, it can replace other multimodal data, such as facial expressions, in addition to multimodal data used in HDECS [43]. This system is not limited to Korean but can be expanded to multiple languages worldwide, allowing for more accurate and segmented emotion classification.

## Figures and Tables

**Table 1:** Parameters of Algorithm 1.

Parameter	Description
Bio	Input data to complementation
result_mode	LSTM model predictions
Interpolated_bios	Complemented biodata list
TotalTime	Total measured time of biodata
post_time	The most recent time value after the current time
pre_time	The most recent time value before the current time
Interpolated_bio	Complemented biodata
dil	Data interval for learning
Interval_bio	The difference between the data post_time and pre_time
Interval_time	post_time–pre_time

**Table 2:** Parameters of Algorithm 2.

Parameter	Description
dfm	Data for model learning
pred_res	Prediction results of the LSTM model using Softmax
weights	Weight of the model
biases	Biases of the model
Metrics_x	Metric of weight and deflection of the x gate
seq	Sequence of LSTM model
Input Gate	Calculating the importance of the input and updating the cell state
Forget Gate	Determine the cell status part to delete
Output Gate	Determine the hidden state of the final output and the next sequence
Cell State	Take the current sequence status and forward it to the next time
Hidden State	Determine the output of the model and the next hidden state

**Table 3:** LSTM model structure for IBI predictions.

Layer	Output
LSTM_1	(None,None,hidden_dim)
Dropout_1	(None,None,hidden_dim)
Dense (Softmax)	(None,hidden_dim)

**Table 4:** Parameters of Algorithm 3.

Parameter	Description
waves	wav files of speech
num_fft	number of fast Fourier transformation
hop_len	hop length between frames
num_mels	number of Mel bins
mel_filters	triangular Mel scale filters
STFT(x, n_fft, hop_len)	short-time Fourier transform function applied to signal x
stft_sp	results of spectrogram from STFT function
mel_sp	Mel spectrogram obtained by applying Mel filter bank to stft_sp
log_mel	the result of transformation mel_sp to Decibel
log_mels	the set of log_mel

**Table 5:** Architecture of the ResNet-50 used in the experiment.

Layer	Filter	Strides	Output
2D CNN	64	(2, 2)	(32, 64, 64)
Batch normalization			(32, 64, 64)
Rectified linear unit			(32, 64, 64)
Max pooling			(15, 31, 64)
Convolution block	(64, 64, 256)	(1, 1)	(15, 31, 256)
Identity block	(64, 64, 256)		(15, 31, 256)
Identity block	(64, 64, 256)		(15, 31, 256)
Convolution block	(128, 128, 512)	(2, 2)	(8, 16, 512)
Identity block	(128, 128, 512)		(8, 16, 512)
Identity block	(128, 128, 512)		(8, 16, 512)
Identity block	(128, 128, 512)		(8, 16, 512)
Convolution block	(256, 256, 1024)	(2, 2)	(4, 8, 1024)
Identity block	(256, 256, 1024)		(4, 8, 1024)
Identity block	(256, 256, 1024)		(4, 8, 1024)
Identity block	(256, 256, 1024)		(4, 8, 1024)
Identity block	(256, 256, 1024)		(4, 8, 1024)
Identity block	(256, 256, 1024)		(4, 8, 1024)
Convolution block	(512, 512, 2048)	(2, 2)	(2, 4, 2048)
Identity block	(512, 512, 2048)		(2, 4, 2048)
Identity block	(512, 512, 2048)		(2, 4, 2048)
Global average pooling			2048
Dense			7
Softmax			1

**Table 6:** Values used for generating the log Mel spectrogram.

Parameter	Value
Window size	4096
Number of FFT	4096
Hop length	3072
Number of Mel filters	64
Padding size	127

**Table 7:** Parameters of Algorithm 4.

Parameter	Description
p_model_	model prediction,
P_model_	P_model_, the set of p_model_
scripts	scripts from speeches
pre_emo	combined emotion prediction from the two models
emo_scripts	scripts that add the emotion sentences

**Table 8:** Prediction conversion to input with CLC.

Emotion	Sentence
angry	그래서 나는 화가 나. (So I’m angry.)
Disgust	으 너무 싫어. (Ugh, I hate it so much.)
fear	그래서 나는 너무 무서웠어. (So I’m so scared.)
happy	그래서 나는 행복해. (So I’m happy.)
Sad	그래서 나는 울었어. (So I cried.)
Surprise	그래서 나는 놀랐어. (So I’m surprised.)

**Table 9:** Formulas for accuracy, categorical cross-entropy, and F1 score evaluation criteria.

Evaluation Criterion	Formula	Functional
Accuracy	$\frac{Correct\;prediction}{Number\;of\;prediction}$	Accuracy measures how accurately it predicted the entire set of predictions.
Categorical cross-entropy	${-\frac{1}{N}\overset{N}{\underset{i=1}{\sum }}\overset{C}{\underset{j=1}{\sum }}t}_{ij}log\left(y_{ij}\right)$	Categorical cross-entropy uses the Softmax function as the activation function, where each output vector represents the probability of belonging to each class, with the total sum equaling 1.
F1 score	$\frac{2}{\frac{1}{Precision}+\frac{1}{Recall}}=\frac{2\times Precision\times Recall}{Precision+Recall}$	The F1 score is the harmonic mean of precision and recall, commonly used when there is data imbalance among classification classes.

**Table 10:** Comparing accuracy, loss, F1 score KLUE/roBERTa-base, and proposed HDECS..

Approach	Accuracy	Loss	F1 Score
KLUE/roBERTa-base	0.81	0.58	0.79
Proposed HDECS	0.90	0.35	0.90

## Data Availability

The data presented in this study are available on request from the corresponding author.
